# Research advances in neuronavigated target localization for repetitive transcranial magnetic stimulation in depression: from standardization to individualized neuromodulation

**DOI:** 10.3389/fnimg.2025.1703198

**Published:** 2026-01-12

**Authors:** Luyang Jiang, Chris Chit Sze Fung, Calvin Pak Wing Cheng

**Affiliations:** 1 Department of Psychiatry, Qiqihar Medical University, Qiqihar, Heilongjiang, China; 2 Department of Psychiatry, Li Ka Shing Faculty of Medicine, The University of Hong Kong, Hong Kong, Hong Kong SAR, China

**Keywords:** depression, individualized neuromodulation, neuronavigated targeting, neuronavigation, repetitive transcranial magnetic stimulation

## Abstract

Depression is a highly disabling mental disorder imposing a substantial burden on global public health. Repetitive Transcranial Magnetic Stimulation (rTMS), as a non-invasive physical treatment modality, demonstrates favorable efficacy and safety in treating depression. However, significant inter-individual variability in treatment response exists, with the reliability of target localization being a key factor influencing efficacy. Traditional non-neuronavigated localization methods (e.g., 5-cm rule, Beam F3 method), while operationally convenient, suffer from limited reliability due to failure to account for individual variations in brain anatomy (e.g., cortical folding) and functional connectivity patterns. In recent years, driven by advances in magnetic resonance imaging (MRI) technology and individualized treatment paradigms, neuronavigated localization methods based on clinical symptom subtypes and patient-specific brain structural/functional connectivity profiles have significantly enhanced localization reliability and personalization, offering novel approaches to overcome efficacy variability. This review systematically summarizes the mechanisms of action and standard treatment protocols of rTMS for depression, with a primary focus on research advances in target localization methodologies. It encompasses the principles, clinical applications, efficacy comparisons, and optimized integration of both non-neuronavigated and neuronavigated techniques across different populations (adolescents, elderly) and symptom subtypes. By critically analyzing current research achievements and challenges, this review aims to provide clinicians with theoretical foundations and practical references for optimizing rTMS treatment protocols, enhancing response rates, and advancing individualized neuronavigated protocols.

## 1 Introduction

Major Depressive Disorder (MDD) is a highly prevalent and disabling common mental disorder characterized by persistent and pervasive low mood, anhedonia (loss of interest or pleasure), and loss of energy, often accompanied by cognitive impairment, somatic symptoms, and suicidal risk, imposing a heavy disease burden on patients, families, and society (Figee et al., 2022). Depression has become the leading cause of mental disability globally, affecting over 300 million people worldwide, and represents a major challenge in public health (Xu et al., 2024).

Regarding treatment strategies, antidepressant medications (e.g., selective serotonin reuptake inhibitors [SSRIs]; serotonin–norepinephrine reuptake inhibitors [SNRIs]) remain the first-line clinical choice. However, their overall response rate is approximately 50–70%, with limitations including slow onset (typically 2–4 weeks), adverse effects (e.g., gastrointestinal disturbances, sexual dysfunction, weight gain), and treatment resistance in some patients (Cipriani et al., 2018; Ghaffari Darab et al., 2020). For Treatment-Resistant Depression (TRD), defined as inadequate response to at least two adequate trials of different antidepressant medications, treatment options are more limited and challenging (Gaynes et al., 2020).

Non-invasive brain stimulation (NIBS) techniques play an increasingly important role in depression management. Repetitive Transcranial Magnetic Stimulation (rTMS), as a non-invasive, safe neuromodulation technique, has become an important complementary or alternative treatment for depression due to its unique mechanism and favorable tolerability (Mikellides et al., 2021). Approved by the US Food and Drug Administration (FDA) for adult depression treatment (Levkovitz et al., 2009), substantial evidence-based data confirms its efficacy and safety (Cao et al., 2023; McClintock et al., 2018; Vonk et al., 2023). Compared to pharmacotherapy, rTMS has a relatively faster onset (symptom improvement often seen within 1–2 weeks), a different and generally milder adverse effect profile (e.g., headache, scalp discomfort at the stimulation site), which are typically transient, beginning during stimulation and generally not persisting for more than a few hours after each session, making it particularly suitable for patients intolerant or unresponsive to medications (Perera et al., 2016). Research has also explored rTMS combined with pharmacotherapy. Multiple studies indicate that combined rTMS and pharmacotherapy shows superior efficacy compared to pharmacotherapy alone or sham rTMS combined with pharmacotherapy in some patients, potentially leading to faster and more effective symptom relief through synergistic effects, especially in moderate-to-severe or treatment-resistant cases (Chen et al., 2017; Cosmo et al., 2021; Wang et al., 2017). However, heterogeneity exists in current combination studies regarding protocol design (e.g., drug type/dose, rTMS parameters) and patient selection; the optimal combination model and synergistic mechanisms require further elucidation through large-scale, high-quality studies (Blumberger et al., 2018).

Despite significant progress in rTMS for depression, a key challenge remains the marked inter-individual variability in treatment response. Traditional non-neuronavigated localization methods (e.g., 5-cm rule), while operationally simple, cost-effective, and widely used clinically, offer limited consistency in targeting the primary stimulation site (dorsolateral prefrontal cortex, DLPFC) due to insufficient consideration of individual variations in brain anatomy (e.g., head size, cortical gyrification) and functional connectivity patterns. This anatomical imprecision is considered a major factor affecting the consistency and optimization of efficacy (Cash et al., 2021c; Gogulski et al., 2023). Studies have found that using traditional methods, the actual stimulation point only partially falls within the target DLPFC region (Herwig et al., 2001; Johnson et al., 2013), undoubtedly diminishing the therapeutic potential of rTMS.

Therefore, improving neuronavigated targeting reliability is a core direction for optimizing rTMS efficacy. In recent years, fueled by the rapid development of neuroimaging (e.g., structural MRI, functional MRI), neuronavigation technology, and the principles of individualized treatment paradigms, various neuronavigated rTMS targeting methods based on individualized brain structural and functional features have emerged and are gradually being applied in clinical practice. These methods aim to overcome the limitations of traditional localization, achieving “individualized” and “anatomically precise” stimulation to maximize therapeutic effects. The novelty of this review lies in its systematic focus on methodological advances in target localization for rTMS treatment of depression. It not only details and evaluates the evolution from non-neuronavigated to neuronavigated localization techniques but also emphasizes the latest evidence on cutting-edge targeting strategies based on clinical symptom subtyping, individualized functional connectivity mapping, and novel guidance techniques (e.g., neuro-cardiac guided). It delves into their underlying mechanisms, clinical efficacy, and existing challenges, providing comprehensive and in-depth references for future research design and clinical practice optimization. This article aims to enhance clinicians‘ and researchers' understanding of the importance and potential of rTMS target localization, offering clues and theoretical support for ultimately realizing individualized neuronavigated protocols for depression.

## 2 Fundamental principles and protocols of rTMS for depression

rTMS is a non-invasive, safe intervention tool. Its fundamental principle utilizes electromagnetic induction, generating a time-varying magnetic field that induces electrical currents in the cerebral cortex, stimulating neuronal depolarization or hyperpolarization, thereby modulating neuronal excitability (Mikellides et al., 2021). Substantial evidence confirms that rTMS effectively improves depressive symptoms with a favorable safety profile (Lefaucheur et al., 2020a). The dorsolateral prefrontal cortex (DLPFC) is the most frequently selected target for rTMS in depression. As a key brain region regulating emotion and cognition, stimulating the DLPFC promotes improvement in depressive symptoms (Lai, 2021; Lefaucheur et al., 2020b; O'Reardon et al., 2007). MDD patients often exhibit decreased functional activity in the left DLPFC and increased activity in the right DLPFC. Therefore, modulating DLPFC function helps improve depressive symptoms (Chen et al., 2017). Based on the traditional understanding of hemispheric specialization in emotional processing, two standard rTMS protocols for depression have been developed: excitatory high-frequency rTMS (HF-rTMS) applied to the left DLPFC and inhibitory low-frequency rTMS (LF-rTMS) applied to the right DLPFC (Abdelrahman et al., 2021). Stimulation parameters are defined as follows: High-frequency stimulation typically refers to frequencies of 5–20 Hz, while LF-rTMS typically refers to stimulation at 1 Hz, though some protocols may use frequencies below 1 Hz (Croarkin and MacMaster, 2019; Lefaucheur et al., 2014). For left-sided HF-rTMS, common clinical protocols often employ a daily pulse count between 1,200 and 1,500, although optimal dosing may vary across individuals and populations (Teng et al., 2017). For right-sided LF-rTMS, Ford et al. compared patients receiving 900 pulses vs. 1,800 pulses and found equivalent clinical efficacy (Ford et al., 2021). Furthermore, Intermittent Theta Burst Stimulation (iTBS), a newer rTMS protocol approved by the FDA in 2018 for treatment-resistant depression, typically has a significantly shorter single-session duration (e.g., 3 min for 600 pulses) than conventional protocols (e.g., 37.5 min for standard 10 Hz rTMS at 3,000 pulses), while demonstrating comparable clinical efficacy (Blumberger et al., 2018). In some accelerated protocols, such as the Stanford Accelerated Intelligent Neuromodulation Therapy (SAINT), multiple iTBS sessions are delivered per day, increasing the total daily treatment time while maintaining the brevity of individual sessions (Cole et al., 2020). This potentially improving patient compliance and treatment accessibility (Blumberger et al., 2018; Chen et al., 2025).

## 3 Localization methods for rTMS intervention in depression

rTMS is widely used in clinical depression treatment globally. How to further enhance its efficacy remains a key research focus, with improvements in target localization methods being a critical direction. Traditional non-precise rTMS localization methods, benefiting from convenience and cost-effectiveness, are extensively applied in clinical practice. With advances in imaging technology and the dissemination of precision medicine concepts, various precise rTMS targeting methods have rapidly developed, often demonstrating superior localization accuracy and treatment outcomes compared to traditional approaches. Selecting the appropriate localization method based on individual patient characteristics holds promise for achieving better therapeutic results.

### 3.1 Advances in non-neuronavigated rTMS localization methods for depression

Currently, clinically prevalent rTMS localization methods without neuronavigation primarily include the standard 5-cm rule and the Beam F3 method based on locating the F3 electrode position via the EEG 10–20 system. The 5-cm rule is a scalp-based technique, while the Beam F3 method is also a scalp-based technique and represents an optimization derived from locating the F3 position using the 10–20 EEG system, utilizing a simplified geometric formula to estimate the F3 electrode site (Beam et al., 2009). These methods are widely used clinically with reasonably reliable efficacy (Corlier et al., 2020; Zheng et al., 2020). Their main advantage lies in relative operational simplicity. However, these methods often result in overly broad DLPFC localization ranges, leading to low anatomic specificity. While the 5-cm rule fails to account for individual head size at all, methods like Beam F3, which incorporate head circumference measurements, offer an improvement. Nevertheless, they still do not address fundamental individual variations in brain size, shape, and cortical gyrification, which are the primary contributors to insufficient DLPFC localization.

#### 3.1.1 Scalp-based targeting methods

The 5-cm localization method, proposed in the 1990s, remains a common approach for DLPFC targeting. This method first identifies the motor hotspot (location eliciting the optimal thumb abductor motor evoked potential—MEP), then moves 5 cm anteriorly along the scalp from this motor hotspot to determine the stimulation target (Pascual-Leone and Hallett, 1994). Numerous studies have demonstrated the efficacy of this method for treating depression (Notzon et al., 2018; Zheng et al., 2020). Despite its simplicity, individual skull anatomical variations often lead to significant targeting errors. Research indicates that using the standard 5-cm rule, the actual stimulation target falls within the DLPFC in only about one-third of patients (Herwig et al., 2001). Furthermore, studies using neuroimaging have confirmed that the 5-cm rule can often result in stimulation sites outside the intended DLPFC region (Johnson et al., 2013). To improve consistency, researchers have derived variants like the 5.5-cm and 6-cm rules (moving the respective distance anteriorly from the motor hotspot). Some studies suggest these variants may target the DLPFC more accurately than the standard 5-cm rule (Gogulski et al., 2023; McClintock et al., 2018). Notably, the DLPFC in the Talairach coordinate system primarily covers Brodmann areas (BA) 46 and 9 (Cash et al., 2021c), and one study found the intersection of BA 46 and BA 9 is located approximately 6.9 cm anterior to the motor hotspot (Ahdab et al., 2010). However, the 6.9-cm method has not yet been widely adopted clinically.

#### 3.1.2 F3 electrode localization based on EEG 10–20 system

To accommodate individual skull variations, a localization method based on the EEG 10-20 system was proposed for optimizing rTMS treatment. This method calculates the position of the F3 electrode on the participant's scalp by measuring head circumference and relevant surface landmarks (e.g., nasion, inion, preauricular points). The F3 position is identified as the intersection point derived from specific measurements: 20% of the nasion-inion distance from the nasion along the midline, and then perpendicularly to the left at a distance of 20% of the total head circumference. As the F3 position approximates the DLPFC location, operators can position the rTMS coil relatively accurately over the DLPFC region to exert antidepressant effects (Beam et al., 2009). However, for operators inexperienced with the 10–20 system, the numerous measurements and calculations can be time-consuming.

#### 3.1.3 Beam F3 method

The Beam F3 method, proposed by Beam et al., simplifies the EEG 10–20 system-based localization by replacing complex measurements with a geometric formula. It calculates the F3 position using head circumference ratios measured between key landmarks: the anterior-posterior coordinate is determined as 35% of the nasion-inion distance anterior from the inion, and the lateral coordinate is 25% of the total inter-tragus distance lateral from the midline.. This reduces operator dependency and measurement time (to ~5 min), maintaining comparable accuracy to the 10–20 system (Beam et al., 2009; Mir-Moghtadaei et al., 2022). The Beam F3 method aims to simplify the 10–20 system-based localization process, reducing measurement and calculation steps to quickly estimate the approximate position of the frontal F3 electrode. Corlier et al. applied Beam F3 for DLPFC localization in rTMS treatment of depression patients and observed significant improvements in cognitive control, supporting the method's validity (Corlier et al., 2020). Trapp et al., through a clinical controlled study, found that targets determined by Beam F3 were located on average about 2.6 ± 1.0 cm anterior-lateral to targets determined by the 5.5-cm rule, demonstrating higher localization reliability compared to the 5.5-cm method, and identifying distinct anatomical targets (Trapp et al., 2020). However, other studies reported no significant statistical difference in antidepressant efficacy between Beam F3 and the 5.5-cm method (Trapp et al., 2023); its clinical value requires further validation through additional trials.

### 3.2 Advances in neuronavigated rTMS localization methods for depression

Neuronavigated rTMS refers to protocols that utilize neuronavigation systems to precisely guide the rTMS coil to a predetermined target location based on the patient's neuroimaging data. The core of this approach is the real-time tracking of the coil position relative to the individual's brain anatomy, typically derived from a structural MRI. While the selection of the stimulation target (e.g., a group-level coordinate vs. a patient-specific site) is a separate decision, neuronavigation is the enabling technology for delivering stimulation with high anatomical precision. It is important to note that although highly personalized target selection generally requires neuronavigation, broader personalization (e.g., choosing between DLPFC and other prefrontal targets) can be attempted without it. Meta-analytic evidence suggests that neuronavigated targeting may confer a modest advantage over non-navigated methods in antidepressant outcomes, potentially by reducing anatomical variability in coil placement (Cash et al., 2021b). However, not all randomized trials have consistently demonstrated superior efficacy, with some reporting comparable outcomes between navigated and non-navigated approaches (Hebel et al., 2021; Li et al., 2020). These discrepant findings may be attributable to variations in sample characteristics, stimulation parameters, or the specific navigated technique employed, underscoring the need for further large-scale, well-controlled comparative effectiveness research.

#### 3.2.1 Target selection based on individual anatomical structure

Utilizing structural MRI, specific coordinate points can be precisely located and set as rTMS stimulation targets (i.e., structural DLPFC targets). Guiding the coil to this structural target using neuronavigation systems achieves superior anatomic consistency than non-navigated methods. Various group-level anatomical coordinates derived from prior studies have been used (see Table 1 for a summary). For example, Mylius et al. set the target coordinates at the junction of BA 9 and BA 46 Mylius et al., (2013). Herbsman et al. averaged the stimulation coordinates of 54 depression patients treated with standard 5-cm rule rTMS, finding an effective stimulation point at (−46, 23, 49) in the Montreal Neurological Institute (MNI) coordinate system, and structural targets (MNI: −46, 23, 49) correlated with better antidepressant outcomes than non-navigated methods (*p* < 0.05) Herbsman et al., (2009). Moreover, structural MRI-guided localization significantly improves targeting reliability. Fitzgerald et al. provided key evidence for the efficacy of structural navigation. In a study of 51 Treatment-Resistant Depression (TRD) patients, neuronavigated rTMS delivered to a standard coordinate (MNI: −46, 45, 38) achieved a 48% response rate, compared to 13% with the 5-cm rule Fitzgerald et al., (2009). This and other targets are compiled in Table 1. Despite variability in MNI coordinates due to anatomical differences, neuronavigation consistently enhances consistency over scalp-based methods Cash et al., (2021b); Fitzgerald et al., (2009).

**Table 1:** Commonly used anatomical targets in neuronavigated rTMS studies for depression.

**Study**	**Target description**	**MNI coordinates (x, y, z)**	**Key findings/rationale**
Herbsman et al. (2009)	Average effective site from 5-cm rule treatments	−46, 23, 49	More lateral/anterior location correlated with better outcome.
Fitzgerald et al. (2009)	Standard anatomical DLPFC target	−46, 45, 38	48% response vs 13% with 5-cm rule in TRD.
Fox et al. (2012)	DLPFC site with strong anti-correlation to sgACC	−38, 44, 26	Functional connectivity-guided; strength predicts efficacy.
Mylius et al. (2013)	Junction of BA 9 and BA 46	(Defined by individual anatomy)	Anatomically precise landmark for DLPFC.

##### 3.2.1.1 CALiper-based precise positioning of the TMS target

Hu et al. recently proposed a precise localization technique as an alternative to full neuronavigation systems: CALiper-based Precise Positioning of the Target (CALIPPOT) (Hu et al., 2024). This method leverages individual MRI for target identification but uses physical calipers for guidance, effectively bridging the gap between non-navigated and fully navigated approaches. The entire localization process can be completed within 10 min: First, three imageable markers (M1, M2, M3) are placed on the subject's scalp. After MRI scanning to locate the target and measure its Euclidean distance to each marker center, caliper distances are set according to these measurements. Two operators then simultaneously place one end of the calipers on the centers of two markers (e.g., M1, M2) and draw arcs on the scalp with the other end; their intersection point is the target stimulation site. Finally, the process is repeated using another pair of markers (e.g., M1, M3) for verification to ensure consistency (Hu et al., 2024). The core value of CALIPPOT lies in achieving millimeter-level accuracy (error <3 mm) while significantly lowering the barrier to neuronavigated localization (costing only about 1/5th of a neuronavigation system). This simple, fast, low-cost technical solution provides a highly promising practical tool for implementing precise rTMS in primary care or resource-limited settings, potentially improving the accessibility of anatomically precise rTMS.

#### 3.2.2 Target selection based on functional neuroimaging

Depression is associated with aberrant changes in brain functional circuits. The foundational principle of functional connectivity-guided targeting is that the antidepressant efficacy of stimulating a specific cortical site is related to the strength of its functional connection with deeper limbic structures implicated in depression pathology (Fox et al., 2012). Research indicates that targets selected based on functional connectivity are thought to be influenced by individual genetics and can exhibit potential stability (Cash et al., 2021b). However, the test-retest reliability and practical robustness of deriving such personalized functional targets, particularly those based solely on the sgACC, have been questioned. Achieving a stable target may require lengthy fMRI scans and sophisticated analysis, posing significant resource challenges (Mir-Moghtadaei et al., 2022; Lynch et al., 2022).

##### 3.2.2.1 Targeting based on clinical symptom subtypes

A prominent line of research aims to define neurophysiological subtypes of depression based on resting-state functional connectivity and map these subtypes to optimal TMS targets and clinical symptom profiles. For instance, Siddiqi et al. categorized depression into a “Distress” subtype (characterized by sadness, anhedonia, suicidality) and an “Anxious Arousal” subtype (characterized by irritability, reduced libido, insomnia). They found that an anterolateral DLPFC target exhibiting negative functional connectivity with the subgenual anterior cingulate cortex (sgACC) effectively improved “Distress” symptoms, whereas posterior and medial prefrontal targets were more effective for the “Anxious Arousal” subtype (Siddiqi et al., 2020). Kaster et al. (2023) grouping symptoms into mood, anxiety, insomnia, and somatic factors, observed that patients in the anxiety symptom group had lower response rates to rTMS targeting the left DLPFC compared to other symptom groups. Drysdale et al. (2017) classified MDD into 4 subtypes based on resting-state functional connectivity abnormality patterns, finding that a subtype dominated by anxiety and insomnia symptoms showed a particularly significant response to rTMS targeting the dorsomedial prefrontal cortex (DMPFC). These seemingly discrepant findings regarding the anxious subtype may be reconciled by considering the different stimulation targets employed. The poorer response reported by Kaster et al. pertained to standard left DLPFC stimulation, whereas the positive response in Drysdale et al.'s study was associated with DMPFC targeting. This underscores the potential importance of matching specific symptom profiles with optimal neurocircuitry targets, rather than applying a one-size-fits-all DLPFC approach.

##### 3.2.2.2 Targeting based on functional connectivity with specific nodes

Another approach involves using a specific, well-validated depression-related brain region as a “seed” to identify the most anti-correlated or connected site within the DLPFC for stimulation. The subgenual anterior cingulate cortex (sgACC) is a key hub for transmitting emotional information between the limbic system and higher-order cognitive structures, exhibiting widespread functional connectivity with cortical and subcortical regions implicated in depressive symptoms (Scharnowski et al., 2020; Yu et al., 2023). Studies found a negative functional connection between the sgACC and DLPFC, and the strength of this connection predicts rTMS efficacy (Weigand et al., 2018). This suggests that identifying the site within the DLPFC exhibiting the strongest negative connectivity with the sgACC as the target may enhance the antidepressant effect of rTMS. Fox et al., in a seminal study, confirmed through retrospective analysis that stronger negative connectivity between the DLPFC target and the sgACC correlated with better rTMS efficacy, leading them to identify a group-level target MNI (−38, 44, 26) with strong sgACC-negative connectivity (Fox et al., 2012). Another study, combining neuroimaging navigation with the Human Brainnetome Atlas (Fan et al., 2016), used sgACC (BA 25) as a seed point for whole-brain resting-state functional connectivity analysis, locating the brain region within the DLPFC with the highest connection strength as the stimulation target MNI (−41, 41, 16). Most of these studies identified universal targets at the group level. Recently, research has also explored finding personalized targets at the individual level, suggesting that individualized precise rTMS may yield superior outcomes (Cash et al., 2021a). However, a recent study raised partial concerns about this approach. Elbau et al., using data from 295 MDD patients to validate the efficacy of individualized treatment based on sgACC-DLPFC connectivity, found that although functional connectivity between sgACC and the stimulated cortex correlated with efficacy, the correlation was weak and possibly confounded by specific breathing patterns (Elbau et al., 2023). It should be noted that Siddiqi and colleagues, in a letter to the editor, discussed methodological concerns regarding the Elbau et al. study, suggesting these might explain the discrepancy with prior literature (Elbau et al., 2023). However, the universality and clinical superiority of this approach require further validation. Crucially, no prospective, randomized controlled trials have yet demonstrated that personalized sgACC-targeting yields superior outcomes compared to group-level functional or anatomical targeting. Recent retrospective evidence even suggests that the distance from the stimulation site to a personalized sgACC-derived target may not consistently predict treatment outcome when standard (non-individualized) targets are used (Gregory et al., 2025; Khosravani et al., 2025).

Besides sgACC, other brain regions may serve as functional connectivity targets. Du et al. found that the strength of functional connectivity between the nucleus accumbens (NAcc) and DLPFC in depression patients predicted the antidepressant and anxiolytic effects of rTMS, suggesting that the point exhibiting the strongest functional connectivity between NAcc (similar to sgACC) and DLPFC could be a potential novel target (Du et al., 2018). Wang et al., by stimulating the point of strongest individualized NAcc-DLPFC functional connectivity, found it effectively alleviated anticipatory anhedonia and improved reward-seeking behavior compared to a sham group (Wang et al., 2021). Furthermore, the anterior pregenual cingulate cortex (pgACC) is another key region in emotion regulation. Altered activity in the pgACC has been implicated in MDD, making it a potential seed region for deriving individualized functional connectivity targets, similar to the sgACC and NAcc. Raij et al. (2023) constructed a Core Network Model (CNM) for emotion, comprising 8 seed points (including amygdala, cingulate cortex), and found that the functional connectivity strength between the CNM and DLPFC correlated with depression severity and rTMS efficacy, providing a new approach for utilizing core network models to screen for individualized optimal targets.

##### 3.2.2.3 fMRI-guided accelerated TMS protocols

The Stanford Neuromodulation Therapy (SNT) protocol, often referred to as SAINT, represents a significant advancement that integrates functional connectivity targeting with an accelerated, high-dose treatment schedule. This protocol uses individualized fMRI to target the left DLPFC site most negatively correlated with the sgACC. Patients then receive multiple iTBS sessions per day over several days (e.g., 10 sessions per day for 5 days). This intensive approach has demonstrated remarkably high response and remission rates in open-label and randomized sham-controlled trials for treatment-resistant depression, leading to its clearance by the US FDA (Cole et al., 2020). The success of SAINT highlights the potential synergy between precise targeting and optimized treatment schedules.

##### 3.2.2.4 Targeted functional network stimulation

Traditional rTMS coil placement often relies on group-average functional maps or scalp-based empirical localization. Research shows that targeting the same anatomical region may inadvertently activate different functional networks in different patients, leading to variability in treatment response (Beynel et al., 2020; Lynch and Liston, 2020). Therefore, traditional methods do not always yield the expected clinical effects. Addressing this issue, Lynch et al. (2022) proposed Targeted Functional Network Stimulation (TANS). This is an automated rTMS coil placement optimization method based on individualized functional networks. Utilizing each individual's unique functional topology and cortical folding pattern, TANS identifies scalp coil positions that maximize stimulation of the target functional network while minimizing off-target effects, achieving true individualized functional network targeting. This novel TMS targeting approach holds promise for delivering more selective stimulation to different depression patients; its clinical utility awaits future validation.

#### 3.2.3 Other individualized neuromavigated approaches: neuro-cardiac-guided TMS

Research indicates the DLPFC-sgACC connection plays a crucial role in depression (Weigand et al., 2018), and prior studies suggest both DLPFC and sgACC can activate the vagus nerve via rTMS stimulation, subsequently lowering heart rate (Iseger et al., 2020; Rossi et al., 2016). This implies that heart rate changes might indirectly reflect the strength of the functional connection between sgACC and DLPFC. Based on this, Iseger et al. proposed a novel localization method: Neuro-Cardiac-Guided TMS (NCG-TMS). A core advantage of NCG-TMS is its relative simplicity, lower cost, and provision of real-time physiological feedback, offering a new pathway for implementing individualized targeting in routine clinical settings (Iseger et al., 2017). This method identifies the site within the DLPFC region that elicits the largest heart rate deceleration as the optimal individualized rTMS target. Its proposal holds promise for simplifying or replacing the complex sgACC-DLPFC connectivity-based localization process. Recent studies have provided further evidence supporting the concept of TMS-induced brain-heart coupling, lending plausibility to this approach (Dijkstra et al., 2024). However, the stability and universality of its efficacy prediction need validation in larger populations, particularly across different depression subtypes, and through direct comparison with the fMRI connectivity-based gold standard (Elbau et al., 2023).

### 3.3 Symptom- and population-specific considerations

As the clinical application of rTMS expands, strategies for target selection and parameter optimization tailored to different populations (e.g., adolescents, elderly) are receiving increasing attention.

#### 3.3.1 Neuronavigated localization and parameter optimization for adolescent depression

Although rTMS and iTBS protocols adapted from adult studies have received regulatory clearance (e.g., FDA) for treating adolescent depression, the ongoing neurodevelopment in this population warrants careful consideration for optimizing treatment. The prefrontal cortex in adolescent depression patients is not fully mature. Traditional standard localization methods (e.g., 5-cm rule) are prone to targeting errors due to differences in skull thickness and brain structure. Navigation-based localization using individual MRI (e.g., CALIPPOT) can significantly enhance DLPFC targeting reliability. Concurrently, stimulation parameters require adjustment: For high-frequency stimulation (10 Hz), the daily pulse count is recommended to be controlled between 900 and 1,200 to avoid over-activating immature neural circuits (Lewis et al., 2024). The mechanism involves precise targeting enhancing the modulation of weaker DLPFC-amygdala functional connectivity in the developing brain, improving emotional impulsivity (Kim et al., 2011).

#### 3.3.2 Localization compensation strategies and protocol selection for geriatric depression

Geriatric depression patients often face anatomical displacement due to brain atrophy. A randomized sham-controlled trial conducted by Jorge's team in patients with vascular depression revealed a negative correlation between age and the severity of frontal lobe gray matter atrophy (Jorge et al., 2008). Studies suggest that when using group-average template-based universal coordinates [e.g., MNI (−46, 23, 49)], a posterior shift of 2–3 mm should be considered to compensate for the anterior displacement effect caused by frontal lobe atrophy (Quinn et al., 2023). Regarding treatment protocol selection, prioritizing low-frequency left-sided stimulation (1 Hz) or intermittent theta burst stimulation (iTBS) protocols, or their combination (e.g., left iTBS + right 1Hz rTMS), has proven better tolerated in elderly patients and effectively improves depressive symptoms and associated cognitive dysfunction (Mi et al., 2022).

#### 3.3.3 Clinical translation challenges of novel targeting paradigms

While novel localization technologies (NCG-TMS, CALIPPOT, TANS) demonstrate significant potential for individualized rTMS delivery, their clinical implementation faces three translational barriers that warrant critical attention: First, technical validation gaps: most emerging techniques lack large-scale RCTs comparing efficacy against neuronavigation or standardized methods. For instance, NCG-TMS's dependency on heart-rate variability requires further validation across depression subtypes (Elbau et al., 2023; Iseger et al., 2021). Second, biological heterogeneity: individual differences in neuroanatomy (e.g., atrophy affecting electric fields) and functional connectivity (e.g., sgACC-NAcc variability) may undermine targeting accuracy benefits, necessitating adjunct biomarkers (Quinn et al., 2023). These barriers highlight an urgent need for standardized validation protocols and streamlined workflows to translate targeting innovations into clinically accessible solutions.

## 4 Summary and outlook

Sixteen years have passed since the US FDA approved rTMS for depression treatment. Extensive clinical trial data has confirmed the reliability and safety of this technology. The most commonly used target localization method in clinical practice remains the 5-cm rule. Despite its limited precision, it is widely applied due to its simplicity and low cost. To accommodate individual skull variations, subsequent methods like the 5.5-cm rule, EEG 10–20 system-based localization, and Beam F3 method were developed. With technological advancement, neuronavigated rTMS is gaining increasing attention. Identifying individualized stimulation targets based on brain functional imaging is considered a promising direction for enhancing efficacy and reducing individual variability. However, its definitive clinical advantage over optimized group-level targets awaits confirmation from large-scale prospective randomized controlled trials. The drive toward personalized targeting is grounded in the understanding that depression is a heterogeneous disorder with individual variations in brain anatomy, functional network organization, and the specific pathophysiological underpinnings of the condition. By accounting for these individual differences, it is theorized that neuromodulation can be more precisely directed to the dysfunctional circuits most relevant to the patient's clinical presentation, thereby maximizing therapeutic potential. Both neuronavigated and non-neuronavigated rTMS treatments have their advantages (Table 2). Future research needs to further explore how to select the most appropriate target localization method based on individual patient symptoms and neuroimaging characteristics to optimize the efficacy and efficiency of rTMS for depression.

**Table 2:** Summary table of target localization methods for rTMS in depression treatment.

**Localization method**	**Principle/operation**	**Advantages**	**Disadvantages**
**Non neuronavigated methods**			
5-cm rule	Locate DLPFC by moving 5 cm anterior from the motor hotspot (evoking thumb abduction MEP)	Simple operation, low cost, widely clinically available	High localization error due to individual skull anatomy (only 1/3 of patients' targets in DLPFC)
Beam F3 method	Rapidly estimate F3 electrode position (≈DLPFC) using a simplified EEG 10–20 system	More accurate than 5-cm rule; fewer measurement steps	Operator experience required; no significant efficacy difference vs. traditional methods
F3 electrode localization (10–20 system)	Calculate F3 position via head circumference measurements	Relatively accurate; reduces impact of anatomical variations	Complex and time-consuming measurements; operator-dependent
**Neuronavigated methods**			
Structural navigation (MRI coordinates)	Set DLPFC coordinates [e.g., MNI(−46, 23, 49)] based on individual MRI	Millimeter-level accuracy; significantly improves targeting precision	MRI-dependent, high cost, complex workflow
Functional connectivity navigation (fMRI)	Localize based on negative functional connectivity strength between sgACC/NAcc and DLPFC [e.g., MNI(−38, 44, 26)]	Personalized target; strong treatment predictability (↑ connectivity → ↑ efficacy)	High fMRI cost; complex data analysis; respiratory artifacts during scanning

This table systematically summarizes the principles, core advantages, and key limitations of non-neuronavigated and neuronavigated localization techniques, encompassing both conventional approaches and emerging individualized technologies. Data synthesized from clinical research evidence aims to serve as a methodological reference for optimizing therapeutic protocols.

In summary, rTMS treatment for depression is undergoing a profound shift from “standardization” toward “individualized precision modulation.” The continuous innovation and optimization of target localization technologies are central to improving response consistency and expanding the beneficiary population. By deepening mechanistic research, strengthening clinical validation, and promoting technology integration and translation, rTMS guided by neuronavigated protocols is poised to play a more efficient and critical role in the future individualized treatment of depression, ultimately benefiting a broader patient population.
